# Genomic Variations in Esophageal Squamous Cell Carcinoma and Esophageal Adenocarcinoma

**DOI:** 10.7759/cureus.45689

**Published:** 2023-09-21

**Authors:** Hatime A Yasar

**Affiliations:** 1 Medical Oncology, Ankara University School of Medicine, Ankara, TUR

**Keywords:** amplification, mutation, genomic alteration, esophageal squamous cell carcinoma, esophageal adenocarcinoma

## Abstract

Objectives

Using a comprehensive dataset derived from the American Association for Cancer Research (AACR) Project Genomics, Evidence, Neoplasia, Information, and Exchange (GENIE), we sought to demonstrate the genetic characteristics of esophageal squamous cell cancer (ESCC) and esophageal adenocarcinoma (EAC).

Methodology

Data were extracted from cBioPortal for cancer genomics (genie.cbioportal.org). Patients with EAC and squamous cell carcinoma were selected. To compare categorical variables, either the chi-square or Kruskal-Wallis test was used. The Benjamini-Hochberg method was applied to correct *P*-values, and consequently, false discovery rate-adjusted *q*-values were computed. When the *q*-value was <0.05, the *P*-value < 0.05 was accepted as statistically significant.

Results

In this study, 1,381 patients with EAC and 312 patients with ESCC were analyzed. Gene alterations were different between the two groups. In EAC, genetic alterations were detected in *ERBB2*, *KRAS*, *SMAD4*, and *TACC3* genes, whereas ESCC exhibited alterations in *CCDN1*, *NFE2L2*, *FGF19*, *FGF3*, *FGF4*, *NOTCH1*, and *CDKN2B* genes.

Conclusions

Notably, this study showed distinct differences in gene alterations between ESCC and EAC, thereby enhancing our understanding of the genetic landscape of these tumors. Further research is required to elucidate the functional implications of these genetic variations to develop targeted therapies that can improve the prognosis of patients with esophageal cancer.

## Introduction

Esophageal cancer is the eighth most common cancer worldwide and the sixth most common cause of death [1]. Esophageal cancer encompasses two primary histological subtypes: squamous cell carcinoma (SCC) and adenocarcinoma (AC). Remarkably, the geographic distribution of esophageal cancer exhibits significant variations. Higher incidence rates were reported in Eastern Asia, Southern Africa, Eastern Africa, Northern Europe, and Southern Central Asia [1]. These geographical differences may be attributed to distinct subhistologies and underlying etiological factors. While the precise risk factors remain elusive, nutritional factors, smoking, alcohol use, gastroesophageal reflux (GER), and previous esophageal diseases such as achalasia, stricture, and Barret’s esophagus are defined as risk factors [2]. Furthermore, ESCC is associated with poor nutritional status, lower socioeconomic status, smoking, and alcohol consumption, whereas EAC is associated with obesity, GER, Barrett's esophagus, and Helicobacter pylori infection [3]. In addition, clinical presentations differ between these two subtypes, with ESCC predominantly located above the tracheal bifurcation (65%), whereas EAC more frequently located below the tracheal bifurcation (94%) [4].

The management of esophageal cancer necessitates multidisciplinary approaches, such as chemoradiotherapy, chemotherapy, and surgery, for locally advanced disease, whereas targeted therapy, immunotherapy, and chemotherapy are therapy options for advanced disease. Despite these treatment options, esophageal cancer has a poor prognosis. The five-year survival rate of esophageal cancer is 14.3%, and ESCC has a worse survival rate than EAC [5]. Furthermore, the treatment strategy varies between ESCC and EAC; human epidermal growth factor receptor 2 (HER2)/ERBB2 status evaluation plays a role in EAC treatment decisions for adding trastuzumab in advanced disease, but there are no recommendations in the guidelines regarding the HER2 evaluation and the addition of trastuzumab for ESCC. In addition, it’s known that ESCC responds better to radiotherapy [6].

The Cancer Genome Atlas (TCGA) project illuminated the distinct nature of ESCC and EAC. Analysis of 164 patients with esophageal cancer as part of the TCGA project clarified two separate disease processes characterized by distinct etiologies and molecular profiles [7]. In this study, our primary objective was to investigate the genetic characteristics of patients with ESCC and EAC using a large and diverse population sample.

## Materials and methods

The AACR Project GENIE

The American Association for Cancer Research (AACR) Project Genomics, Evidence, Neoplasia, Information, and Exchange (GENIE) is a multi-phase, multi-year, international data-sharing project that aims to catalyze precision cancer medicine [8-10]. In the context of this study, the data used were derived from the AACR Project GENIE. Version 13.1 of the public cohort was used [10,11]. Approximately 150,000 patients were included in this dataset. Permissions regarding patient privacy were obtained from the local institutional review board (IRB) of the participating institutions. The consortium was assembled through two legal constructs: a master participation agreement (MPA) and a data use agreement (DUA). The MPA also requires that each institution share data in a manner consistent with patient consent and center-specific IRB policies. The exact approach varies by institution but largely falls into one of three categories: IRB-approved prospective patient consent to sharing, retrospective IRB waivers, and IRB approvals of GENIE-specific research proposals [10]. Anonymized data were shared by the institutions. The GENIE Project database includes Clinical Laboratory Improvement Amendments (CLIA)/International Organization for Standardization (ISO)-certified genomic data, which are routinely collected across multiple international institutions [10]. Detailed information can be found on cbioportal.org.

Data were extracted from cBioPortal for cancer genomics (genie.cbioportal.org). Patients with esophageal AC and SCC were selected, as determined by the OncoTree cancer type taxonomy.

Statistical analysis

In the descriptive analysis of the data, continuous variables were given using the median and interquartile range (IQR), whereas categorical variables were summarized as percentages. To compare the categorical variables, either the chi-square or Kruskal-Wallis test was used. The Benjamini-Hochberg method was applied to correct *P*-values, and consequently, false discovery rate-adjusted *q*-values were computed. When the *q*-value was <0.05, the *P*-value < 0.05 was accepted as statistically significant.

## Results

A total of 1,450 samples from 1,381 patients with EAC and 327 samples from 312 patients with ESCC were analyzed using the AACR Project GENIE data version 13.1 public cohort.

**Figure 1:**
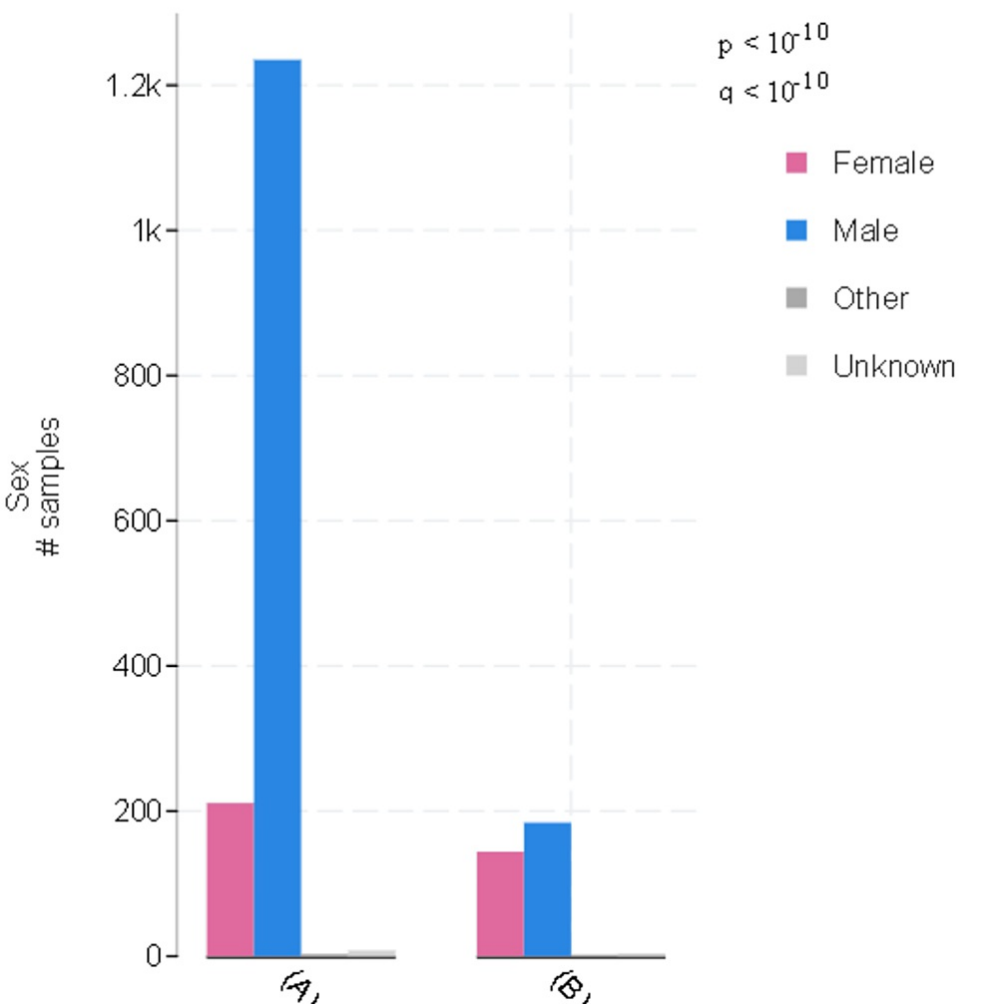
Gender distribution in the EAC and ESCC groups: (A) EAC (N = 1,442); (B) ESCC (N = 324). Blue bars represent males (EAC, *N* = 1,233, 85.09%; ESCC, *N* = 182, 55.8%), and pink bars represent females (EAC, *N* = 209, 14.2%; ESCC, *N* = 142, 43.56%). The difference between the groups was statistically significant (*P *< 0.01). EAC, esophageal adenocarcinoma; ESCC, esophageal squamous cell carcinoma

In the EAC group, 14.42% (209) of the samples were female and 85.09% (1,233) were male. In the ESCC group, 43.56% (142) were female and 55.84% (182) were male. Notably, there was a statistically significant difference in gender distribution between the two groups (*P *< 0.01) (Figure 1).

The majority of EAC (1,235, 85.23%) and ESCC (236, 72.39%) patients were Caucasian. Sampling was performed from the primary site in 66.1% (959) of patients with EAC and 73.1% (239) with ESCC. Data centers are shown in Table 1.

**Table 1:** Data centers. Data are represented as numbers (*N*) and percentages (%).

Center	EAC, *N* (%)	ESCC, *N* (%)
Memorial Sloan Kettering Cancer Center	482 (34.9%)	110 (35.3%)
Dana-Farber Cancer Institute	468 (33.9%)	96 (30.8%)
Providence Health and Services Cancer Institute	103 (7.5%)	11 (3.5%)
The University of Texas MD Anderson Cancer Center	58 (4.2%)	16 (5.1%)
Duke Cancer Institute, Duke University Health System	51 (3.7%)	17 (5.4%)
Wake Forest Baptist Medical Center, Wake Forest University Health Sciences	51 (3.7%)	6 (1.9%)
Yale Cancer Center, Yale University	24 (1.7%)	11 (3.5%)
Vanderbilt-Ingram Cancer Center	23 (1.7%)	4 (1.3%)
University of California, San Francisco	20 (1.4%)	12 (3.8%)
Princess Margaret Cancer Centre, University Health Network	20 (1.4%)	13 (4.2%)
The Herbert Irving Comprehensive Cancer Center, Columbia University	19 (1.4%)	2 (0.6%)
Netherlands Cancer Institute, on behalf of the Center for Personalized Cancer Treatment	18 (1.3%)	3 (1%)
Institut Gustave Roussy	12 (0.9%)	3 (1%)
Johns Hopkins Sidney Kimmel Comprehensive Cancer Center	12 (0.9 %)	0
Vall d’Hebron Institute of Oncology	12 (0.9 %)	5 (1.6%)
Swedish Cancer Institute	8 (0.6%)	3 (1%)

**Figure 2:**
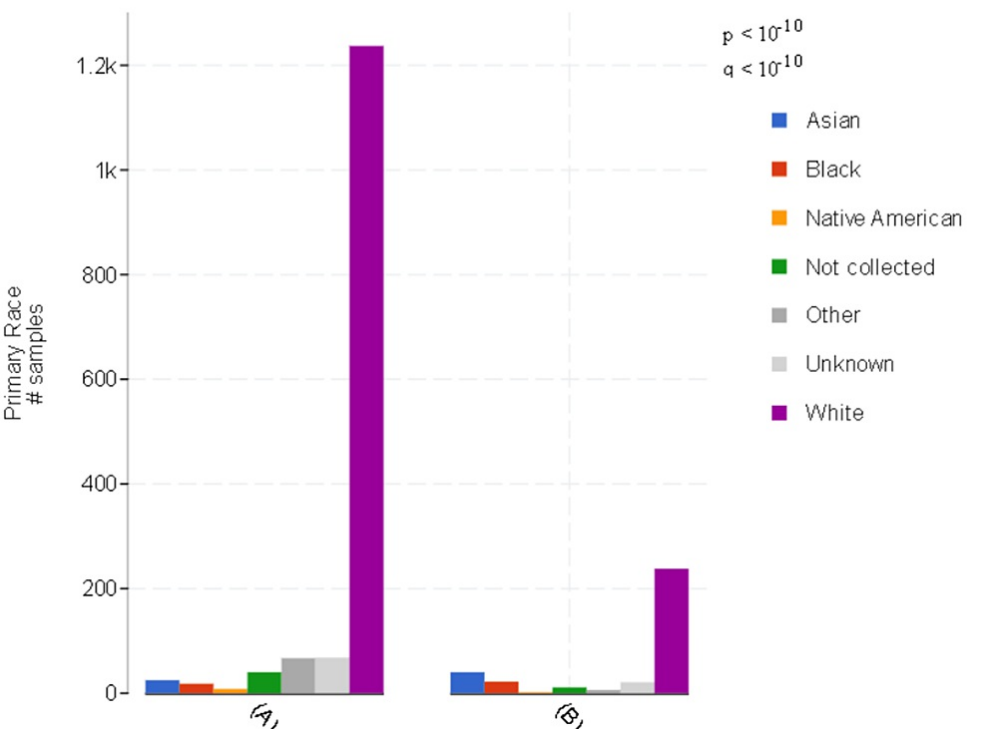
Race distribution in the (A) EAC and (B) ESCC groups. White race: EAC (*N* = 1,235, 85.23%); ESCC (*N* = 236, 72.39%). The difference between the groups was statistically significant (*P *< 0.01). EAC, esophageal adenocarcinoma; ESCC, esophageal squamous cell carcinoma

There was a statistically significant difference in the distribution of racial background between ESCC and EAC patient groups (*P *< 0.01) (Figure 2).

The median age (minimum-maximum) for EAC patients was 63 (35-88) years, while for ESCC, it was 65 (39.5-88) years. There was a statistically significant difference in median age between EAC and ESCC groups (*P *< 0.01) (Table 2).

**Table 2:** Clinical and genomic features of EAC and ESCC. Data are represented as numbers (*N*) and quartiles (q1-q3). The difference between groups was statistically significant (*q *< 0.05). EAC, esophageal adenocarcinoma; ESCC, esophageal squamous cell carcinoma

	EAC	ESCC	*P*-value
Median age (years) (minimum-maximum)	63 (35-88)	65 (39.5-88)	<0.01
Mutational count, median (q1-q3)	7 (4-10)	8 (5-11)	<0.05
Fraction of genome alteration, median (q1-q3)	0.18 (0.05-0.35)	0.29 (0.14-0.5)	<0.05

In terms of mutational count, the median (q1-q3) was 7 (4-10) for EAC and 8 (5-11) for ESCC. The median (q1-q3) for the fraction of genome alteration was 0.18 (0.05-0.35) for EAC and 0.29 (0.14-0.5) for ESCC. Both the fraction genome alterations and mutational count were significantly different between the two groups (*P *< 0.05) (Table 2).

**Figure 3:**
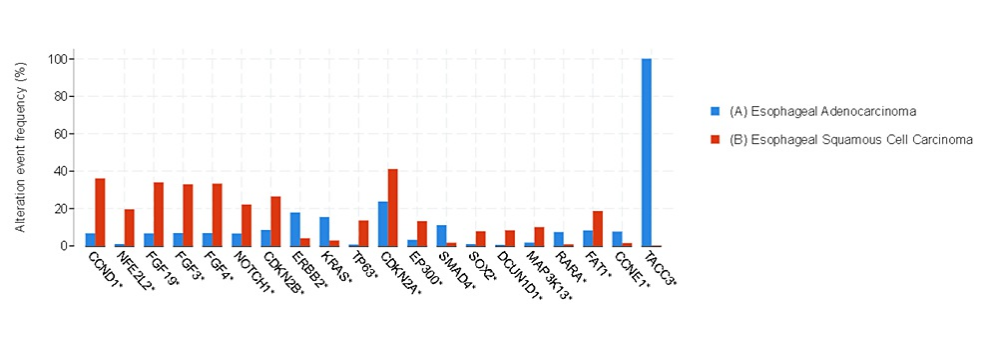
Gene alterations (mutation, structural variants, and copy number alteration) with the most significant value. (A) Blue bars represent EAC, and (B) red bars represent ESCC. Data are represented as percentages (%). ^*^The difference between the groups was statistically significant (*q *< 0.01). EAC, esophageal adenocarcinoma; ESCC, esophageal squamous cancer

*ERBB2*, *KRAS*, *SMAD4*, and *TACC3* gene alterations were predominantly observed in EAC, whereas *CCDN1*, *NFE2L2*, *FGF19*, *FGF3*, *FGF4*, *NOTCH1*, and *CDKN2B* gene alterations were mostly observed in ESCC. Differences in gene alterations between the two groups were significant (*q *< 0.01) (Figure 3). 

**Figure 4:**
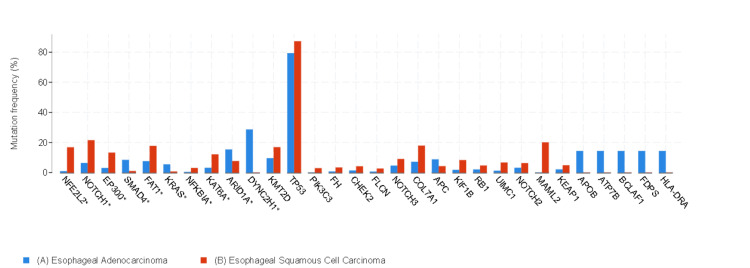
Gene mutations with the most significant value. A) Blue bars represent EAC, and (B) red bars represent ESCC. Data are represented as percentages (%). ^*^The difference between the groups was statistically significant (*q* < 0.05). EAC, esophageal adenocarcinoma; ESCC, esophageal squamous cancer

In ESCC, the most frequently mutated genes were *NFE2L2*, *NOTCH1*, *EP100*, *FAT1*, *NFKBIA*, *KAT6A*, and *KMT2D*. In EAC, the genes that exhibited the highest mutation rates were *SMAD4*, *KRAS*, *ARID1A*, and *DYNC2H1*. The differences in mutated genes between the two groups were significant (*q *< 0.05) (Figure 4).

**Figure 5:**
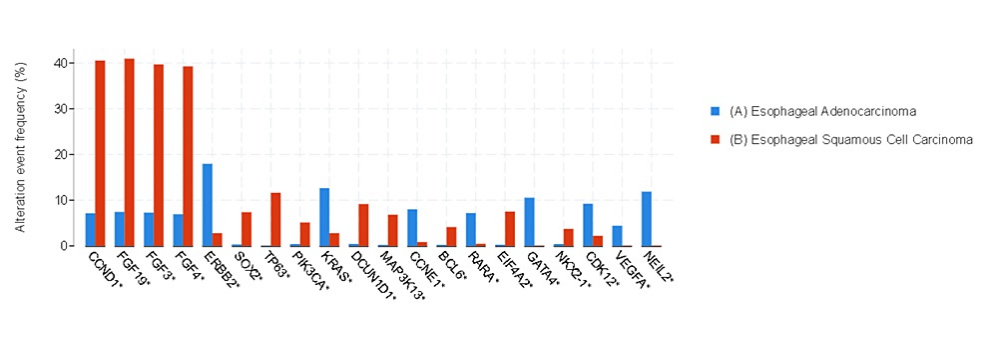
Copy number amplification with the most significant value. (A) Blue bars represent EAC, and (B) red bars represent ESCC. Data are represented as percentages (%). ^*^The difference between the groups was statistically significant (*q* < 0.01). EAC, esophageal adenocarcinoma; ESCC, esophageal squamous cancer

Amplifications of *CCDN1*, *FGF19*, *FGF3*, *FGF4*, *SOX2*, *PI3KA*, and *MAP3K13* were observed more frequently in ESCC, whereas amplifications of *ERBB2*, *KRAS*, *CCNE1*, *RARA*, *GATA4*, and *VEGFA* were more common in EAC. The differences in gene amplification between the two groups were significant (*q *< 0.01). *CCDN1* amplification was detected in 40.4% (107) of ESCC, whereas *ERBB2* amplification was found in 17.8% (205) of EAC (Figure 5).

## Discussion

The molecular characteristics of tumors are important for precision therapy, especially in tumors with poor survival, and are also important for achieving improved outcomes. In this study, the genomic distinctions between EAC and ESCC were highlighted, and it is worth noting that this study represents one of the largest cohorts of patients with EAC to date.

In this study, while both EAC and ESCC were more prevalent among males, there was a notable difference in the male-to-female ratio, with ESCC having a ratio of 1.2 and EAC having a ratio of 6. In addition, in this cohort, it was found that ESCC was more frequently diagnosed in Asian and Black populations than in EAC. This is consistent with the literature, which has also reported higher male-to-female ratios, albeit with some variations [12].

The TCGA Research Network Analysis working group reported genomic analysis of 162 patients with esophageal cancer. There were 90 patients with ESCC and 72 patients with EAC in the TCGA study. *TP53*, *NFE2L2*, *MLL2*, *ZNF750*, *NOTCH1*, and *TGFBR2* were significantly mutated genes in ESCC. In EAC, *TP53*, *CDKN2A*, *ARID1A*, *SMAD4*, and *ERBB2* were reported to be significantly mutated genes in the TCGA study [7]. Gao et al., Cheng et al., Agrawal et al., Song et al., and Sawada et al. also reported similar results for ESCC, and Dulak et al. reported similar results for EAC with the TCGA study [9,13-17]. In this study, *NFE2L2*, *NOTCH1*, *EP100*, *FAT1*, *NFKBIA*, *KAT6A*, and *KMT2D* were the most mutated genes in ESCC; *SMAD4*, *KRAS*, *ARID1A*, and *DYNC2H1* were the most mutated genes in EAC, similar to the literature.

Frequent genomic amplifications of *CCND1*, *SOX2*, and/or *TP63* were observed in ESCC; however, EAC showed frequent amplifications of *ERBB2*, *VEGFA*, *GATA4*, and *GATA6* in the TCGA study [7]. Regarding genomic amplification, it was noted that *CCDN1*, *FGF19*, *FGF3*, *FGF4*, *SOX2*, *PI3KA*, and *MAP3K13* were frequently amplified in ESCC, whereas *ERBB2*, *KRAS*, *CCNE1*, *RARA*, *GATA4*, and *VEGFA* amplifications were more common in EAC in this study, as similar to the literature.

In the literature, HER2 overexpression was found in 11% to 29% of EAC [18-22]. In this study, EAC showed high *ERBB2* amplification, similar to the literature. Yoon et al. and Plum et al. showed significantly better overall survival in patients with EAC [20,23]. Cui et al. reported that impaired *NFE2L2* function was related to impaired tumor suppressor function and NFE2L2 mutations were related to worse overall survival in patients with ESCC [24]. Essakly et al. reported that *KRAS *amplification was 17.1% and associated with poorer survival in patients with EAC [25]. In this study, the relationship between gene alterations and survival could not be evaluated because data were not available.

It is important to acknowledge the limitations of this study, including its retrospective design, lack of comprehensive patient clinical and genomic characteristics (tumor stage, lymph node, grade, comorbid features, and mRNA and miRNA sequencing), limited representation of Asian populations, and the absence of treatment and survival data. Further studies should explore the relationship among genomic characteristics, treatment outcomes, and survival rates in these distinct esophageal cancer types.

## Conclusions

In conclusion, this study highlighted the substantial genomic distinctions between ESCC and EAC within a significant patient population. These two esophageal cancer types exhibit distinct etiological, clinical, and genomic characteristics. It’s important to emphasize the identification and comprehension of these genomic changes to broaden treatment options. Recognizing these genomic alterations provides newer options for developing more precise and effective therapeutic strategies. Further studies are required to define detailed genomic alterations and their relationship with treatment and survival.
